# Notes on Two Cases of “Shoulder Presentation”

**Published:** 1863-01

**Authors:** Morton M. Eaton

**Affiliations:** Peoria, Ill.


### TWO CASES OF "SHOULDER PRESENTATION."

By MORTON M. EATON, M. D., Peoria, Ill.

These cases are reported that encouragement may be given to those who advocate "that the care of a lady in confinement should never be entrusted to any save a well educated physician."

CASE 1ST.—Was called, March 6, 1862, to visit Mrs. R. I found her in the care of a "female physician," who reported that Mrs. R. had been in labor over two days; that this was her first confinement; that for the first twenty-four hours the labor went on well, then the pains became weak, at which time warm teas were given, which after a time brought on strong pains, and the feminine M. D. thought all was right and promised speedy deliverance; but, much to her amazement, in about three hours she discovered a hand of the child in the vagina. This frightened her. But she had heard of the hands coming by the side of the head sometimes, and so she told the people that the hand had come down, but that this would only delay matters, and that if they would be easy the case would terminate favorably in due time. Mr. R. did wait till his wife had been in labor nearly two days, when he became alarmed and dissatisfied, and proposed to have a *Regular Physician* called, which Mrs. M. D. reluctantly assented to.

On reaching the residence of my new patient, I was met by Mr. R., who appeared much alarmed, and wished me to excuse their having had a Mrs. ———, which he said should be the last time one should enter his house, and told me if possible to save his wife and child, and that any amount I asked should be paid. He being a man of wealth, I scolded a little about his trying to save five dollars by getting a female, and told him he would lose fifty dollars by it and possibly something worse.

Thinking I had given him a good pill to digest, I entered the house, and learned from Mrs. Dr. that Mrs. R. had not had any pains for five or six hours, and she said I must give something to bring them on again. I told her I would see our patient first, if agreeable. I was accordingly conducted to the sick room. I found Mrs. R. almost pulseless, in a very warm, close room. I ordered the room ventilated, and the face and hands bathed in water and alcohol. Soon after I made a vaginal examination; found the right hand and fore arm of the child external to the mother and the shoulder closely impacted in the pelvis; by auscultation no pulsations of the fœtal part were discovered. I gave my patient, Opii Pulv. gr. ij; Brandy (French) f℥ ss. M.—and sought Mr. R., to whom I stated the facts of the case, and that a resort to artificial means was absolutely necessary; and that I thought the child was already dead from the long-continued pressure to which it had been subjected, but I thought the mother could still be saved. I told him the child should have been turned soon after the waters broke, which I thought would have saved the life of the child and would have been much less dangerous. I told him that turning then seemed almost impossible; but that I would attempt it, and if I failed, instruments would have to be used. I asked him if he wanted any other physician to see his wife, and he said he did not, but wanted I should do just as I thought best. I accordingly proceeded to attempt to turn, which I accomplished by using some patience and perseverance, and delivered the mother of a large, fine, though dead, female child. The mother by close attention recovered well.

I charged an appropriate bill, which was cheerfully and promptly paid; and as they and all their immediate friends have since employed me entirely, I can't complain; still the case was a bad one for *Mrs. M. D.*

Case 2nd.—This was an unusual case on account of the ease with which I introduced my hand into the vagina and uterus, and the facility with which I corrected the mal-presentation, and shows that we may at times feel that we are of service to the suffering, although often unappreciated.

I was called Dec. 12th to see Mrs. D., in confinement with the fourth child. I learned that she usually had a very easy time. When I made my first vaginal examination of her case I could discover no presentation, although the os uteri was largely dilated. After an hour (during which time she had hard pains, indicating labor in the first stage,) I again examined, and even by passing two fingers into the vagina and reaching as far as possible, nothing of the child could be felt. I examined the uterine globe, and being satisfied a child was there somewhere, I resolved to wait an hour longer and then make a more thorough examination. Accordingly, after waiting, the waters broke, when I prepared my hand and arm and introduced my hand into the vagina, when I was able to reach one shoulder, directly over the centre of the pelvis and the os occipitalis, lying high on the left ilium, and in the absence of a pain I advanced my hand, opened the fingers and seizing the head of the child, much to my own surprise, I brought the head to the superior strait in the second position, and maintained my hold till a pain came on, when I withdrew my hand, and as pains continued good and bearing down I made no further examination for some time, and when I did so I found the head had progressed a little, but seemed to be pressing too much on the left ilium. I pressed it to the right and labor progressed naturally, and I received a large healthy and live girl.

Now, Dec. 17th, both mother and babe are doing nicely. So much for timely interference.

In closing I will acknowledge that when I introduced my hand in the womb I supposed I should have to deliver by the feet, and I was astonished myself that I could bring down the head, (an undertaking that I never before tried, although I have often delivered by the feet for various reasons.) I think that it is seldom that the head could be brought down even if attempted.

